# Supplementary material for: MetaRibo-Seq measures translation in microbiomes
Source: Nat Commun. 2020 Jun 29;11:3268. doi: 10.1038/s41467-020-17081-z (PMC7324362; doi:10.1038/s41467-020-17081-z)
Supplement: Supplementary file 10 — Supplementary Data 7 [file 41467_2020_17081_MOESM10_ESM.zip › File2/Confidence_High_Taxonomy/253291_out.krona.html]

Javascript must be enabled to view this page.

members
magnitude
magnitudeUnassigned
count
unassigned
taxon
rank

253291\_out

11

superkingdom
11
2

976
phylum
11

class
11
200643

order
11
171549

815
11
family

816
11
genus

246787

SRS097920\_contig\_number\_2605
species
1


SRS893366\_contig\_number\_23584
818
1
species


SRS014948\_contig\_number\_6323SRS020869\_contig\_number\_22129SRS045645\_contig\_number\_29906SRS1041039\_contig\_number\_12
329854
species
4

1
species
162156

SRS893230\_contig\_number\_7794

species
1

SRS076756\_contig\_number\_11
1262754

1739298

SRS1055056\_contig\_number\_8956SRS143070\_contig\_number\_16476
2
species

species
1
46506

SRS013687\_contig\_number\_10898
